# Treatment of post-cholecystectomy biliary strictures with fully-covered self-expanding metal stents – results after 5 years of follow-up

**DOI:** 10.1186/s12876-019-1129-3

**Published:** 2019-12-12

**Authors:** Andrea Tringali, D. Nageshwar Reddy, Thierry Ponchon, Horst Neuhaus, Ferrán González-Huix Lladó, Claudio Navarrete, Marco J. Bruno, Paul P. Kortan, Sundeep Lakhtakia, Joyce Peetermans, Matthew Rousseau, David Carr-Locke, Jacques Devière, Guido Costamagna, for the Benign Biliary Stenoses Working Group

**Affiliations:** 1 Fondazione Policlinico Universitario A. Gemelli IRCCS, Digestive Endoscopy Unit, Rome, Italy; 2 Università Cattolica del Sacro Cuore, Centre for Endoscopic Research Therapeutics and Training (CERTT), Rome, Italy; 3 Gastroenterology and Therapeutic Endoscopy, Asian Institute of Gastroenterology, Hyderabad, India; 4 Service de Gastroentérologie et d’Endoscopie Digestive, Hôpital Edouard Herriot, Lyon, France; 5 Medizinische Klinik, Evangelisches Krankenhaus Düsseldorf, Düsseldorf, Germany; 6 Unidad de Endoscopia, Servicio de Aparato Digestivo, Hospital Universitari Doctor Josep Trueta, Girona, Catalunya Spain; 7 Servicio de Endoscopía, Clínica Alemana de Santiago. Jefe de Departamento de Cirugia, Clinica Santa Maria, Santiago, Chile; 8 Maag-, Darm- en Leverziekten, Erasmus Universitair Medisch Centrum, Rotterdam, The Netherlands; 9 Division of Gastroenterology, Centre for Therapeutic Endoscopy and Endoscopic Oncology, St. Michael’s Hospital, Toronto, Ontario Canada; 10 Boston Scientific Corporation, Marlboro, Massachusetts United States; 11 The Center for Advanced Digestive Care, Weill Cornell Medicine, New York Presbyterian Hospital, New York, USA; 12 Gastro-Entérologie et d’Hépato-Pancréatologie, Universite Libre de Bruxelles Hôpital Erasme, Brussels, Belgium

**Keywords:** Benign biliary stricture, Cholecystectomy, V fully-covered self-expanding metal stents

## Abstract

**Background:**

Endoscopic treatment of post-cholecystectomy biliary strictures (PCBS) with multiple plastic biliary stents placed sequentially is a minimally invasive alternative to surgery but requires multiple interventions. Temporary placement of a single fully-covered self-expanding metal stent (FCSEMS) may offer safe and effective treatment with fewer re-interventions. Long-term effectiveness of treatment with FCSEMS to obtain PCBS resolution has not yet been studied.

**Methods:**

In this prospective multi-national study in patients with symptomatic benign biliary strictures (*N* = 187) due to various etiologies received a FCSEMS with scheduled removal at 6–12 months and were followed for 5 years. We report here long-term outcomes of the subgroup of patients with PCBS (*N* = 18). Kaplan Meier analyses assessed long-term freedom from re-stenting. Adverse events were documented.

**Results:**

Endoscopic removal of the FCSEMS was achieved in 83.3% (15/18) of patients after median indwell of 10.9 (range 0.9–13.8) months. In the remaining 3 patients (16.7%), the FCSEMS spontaneously migrated and passed without complications. At the end of FCSEMS indwell, 72% (13/18) of patients had stricture resolution. At 5 years after FCSEMS removal, 84.6% (95% CI 65.0–100.0%) of patients who had stricture resolution at FCSEMS removal remained stent-free. In addition, at 75 months after FCSEMS placement, the probability of remaining stent-free was 61.1% (95% CI 38.6–83.6%) for all patients. Stent or removal related serious adverse events occurred in 38.9% (7/18) all resolved without sequalae.

**Conclusions:**

In patients with symptomatic PCBS, temporary placement of a single FCSEMS intended for 10–12 months indwell is associated with long-term stricture resolution up to 5 years. Temporary placement of a single FCSEMS may be considered for patients with PCBS not involving the main hepatic confluence.

**Trial registration numbers:**

NCT01014390; CTRI/2012/12/003166; Registered 17 November 2009.

## Background

Endoscopic stenting is a recognized [1, 2] and increasingly adopted treatment modality for benign biliary strictures (BBS). Endotherapy of BBS may include any combination of ductal dilation with biliary balloons and extended duration endoscopic stenting. The latter involves placement of multiple plastic stents (MPS) and subsequent MPS exchanges every 3 to 4 months for approximately 1 year, or temporary placement of a fully covered self-expanding metal stent (FCSEMS). These endoscopic treatment modalities are all focused on re-establishing luminal patency.

Post-operative BBS most typically result from bile duct injury during cholecystectomy, with post-cholecystectomy biliary strictures (PCBS) occurring in 0–0.6% of laparoscopic cholecystectomy cases [3–5]. PCBSs may cause chronic cholestasis and jaundice, recurrent cholangitis, and secondary biliary cirrhosis, all of which can lead to serious and even fatal outcomes.

Endotherapy of post-operative BBS using a MPS technique was first reported in the late 1980s and 1990s using one or two biliary plastic stents [6–8]. The concept of using the MPS approach in a more aggressive manner until complete disappearance of the stricture was first reported in 2001 in a large series of 45 patients with BBS, of which 38 were PCBS [9]. Outcomes of endotherapy for these patients after mean follow-up of 13.7 years (range 11.7–19.8 years) was subsequently published in 2011 [10]. Results from endotherapy using MPS in PCBS were also reported from other studies [11–13]. The MPS therapeutic approach has high success rates, but requires multiple interventions and is technically demanding. Stricture dilation is step-wise in the MPS treatment approach. Increased number and/or diameter of plastic stents are inserted for about 3 months, then exchanged up to a cumulative stenting duration of approximately 1 year.

**Fig. 1:**
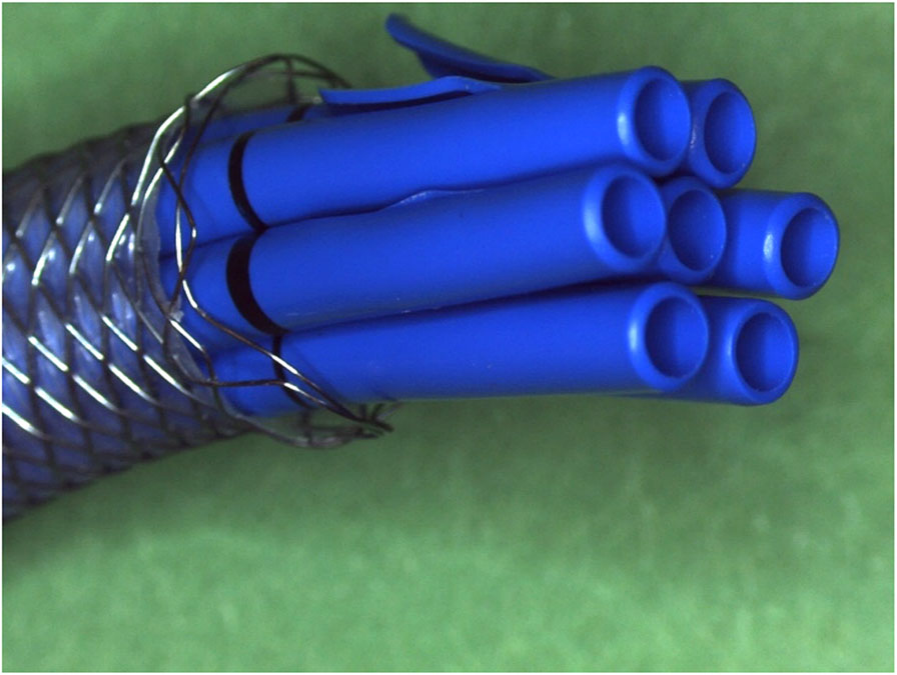
A 10 mm diameter Fully Covered Self-Expandable Metal Stent corresponds to seven 10 Fr plastic stents

FCSEMSs, which are mounted on a delivery system with a diameter comparable to the diameter of one plastic stent, expand to a diameter similar to that of the largest bundle of MPS (seven 10 Fr plastic stents) after approximately 1 year of multi-procedure treatments (Fig. 1). However, use of FCSEMS for endoscopic treatment of PCBS to date is considered investigational [1, 2]. This approach is gaining acceptance, but long-term follow-up is lacking. We previously reported the results of a prospective, non-randomized study assessing the placement of a biliary FCSEMS (WallFlex Biliary RX Stent; Boston Scientific, Natick, MA) with intended indwell for 6–12 months, in 187 patients with BBS secondary to chronic pancreatitis, or anastomotic BBS after orthotopic liver transplantation, or caused by bile duct injury during cholecystectomy (PCBS). Endoscopic removal of the FCSEMS was achieved in 83.3% (15/18) of patients with PCBS, after median indwell of 10.9 months (range 0.9–13.8 months); of these, a stent-in-stent removal technique was required in 1 patient without complications. In the remaining 3 patients (16.7%) with PCBS, the FCSEMS spontaneously migrated and passed without complications. Stricture resolution at the end of FCSEMS indwell was achieved in 72% (13/18) of patients. The present report provides long-term efficacy up to 5 years after FCSEMS removal in the subgroup of patients with PCBS.

## Methods

### Study design

The design of this study (NCT01014390 and CTRI/2012/12/003166) has been described previously [14]. The trial was approved by the Ethics Committee at each participating center and all patients provided written informed consent.

Adverse events were reported by the investigators and categorized as related or unrelated to the study stent, the stenting procedure, or the study stent removal procedure. An independent medical reviewer (DCL) adjudicated the reported relatedness for all deaths and serious adverse events (SAEs). This independent review was carried out blinded to the study site.

### Patients

Eligibility for the index FCSEMS placement procedure included patients ≥18 years old who underwent cholecystectomy and who were indicated to have endoscopic retrograde cholangiopancreatography (ERCP) and stent placement for treatment of BBS. Indications for ERCP were one or more of a symptomatic bile duct stricture, obstructive jaundice, persistent cholestasis and acute cholangitis. The FCSEMS was placed either in exchange of previously placed plastic stent(s), or as initial treatment of a de novo biliary stricture as confirmed by ERCP.

Principal exclusion criteria were history of hepatectomy or liver transplantation, strictures within 2 cm of the hilum, prior biliary SEMS, bile duct perforation or fistula, suspected bile duct ischemia, symptomatic duodenal stenosis, biliary stricture of malignant etiology or benign etiology other than cholecystectomy-related bile duct injury, and strictures too tight to be dilated sufficiently to pass the stent delivery system.

### Stenting and stent removal

The Fully-Covered WallFlex Biliary RX Stent is cleared in most countries outside of the United States for palliation of biliary obstructive symptoms caused by malignant biliary strictures and for treatment of BBS. In the United States it is cleared for palliation of malignant biliary strictures, but the BBS treatment clearance is restricted to BBS secondary to chronic pancreatitis.

**Fig. 2:**
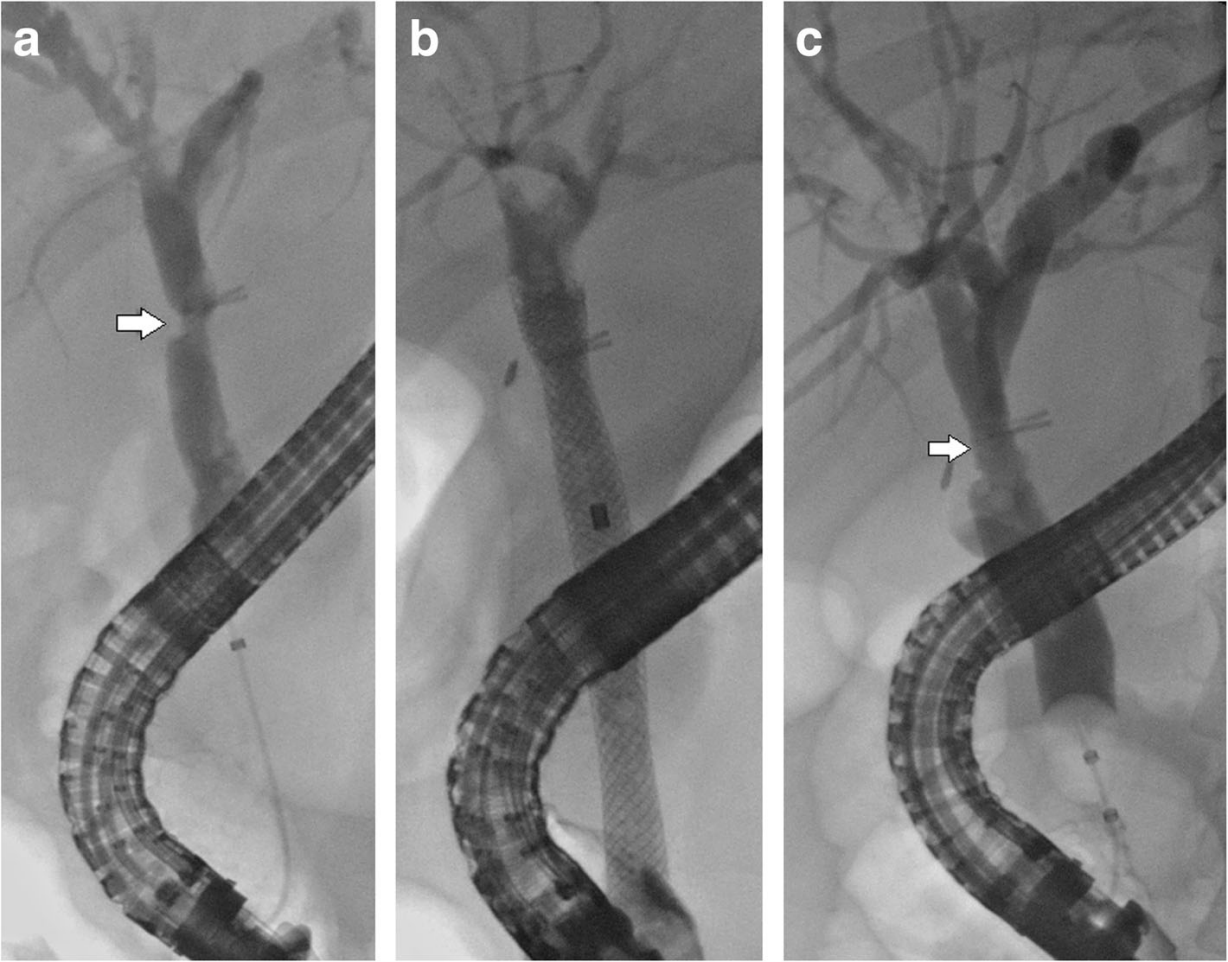
**a** Cholangiogram showing a post cholecystectomy stricture at > 2 cm from the main hepatic confluence near the laparoscopic clips (arrow). **b** Fully Covered Wallflex stent is deployed. **c** Stricture resolution (arrow) after Wallflex removal

The stent is available in 5 sizes, 8 × 60 mm, 8 × 80 mm, 10 × 40 mm, 10 × 60 mm, and 10 × 80 mm. It is made of radiopaque Nitinol wire and a silicone covering. Both ends are flared. The delivery system is 8.5F. It is customary to deploy the stent over an 0.035 in. wire under fluoroscopic guidance (Fig. 2).

The planned FCSEMS indwell duration was 10–12 months, which is a stenting duration similar to what is typical in the MPS treatment. The distal (duodenal) end of the stent has a retrieval loop to facilitate stent removal. Most often, the stent is removed with rat tooth forceps grasping the retrieval loop or by placing a snare over the distal end of the stent.

### Assessments

The goal of PCBS treatment by temporary placement of a FCSEMS is for patients to remain stent-free for an extended period of time without interventions. The fraction of stent-free patients 5 years after FCSEMS indwell and reintervention rates were analyzed.

As previously reported [14] other key assessments were stricture resolution at the end of FCSEMS indwell, stricture recurrence, and adverse events. Stricture resolution was defined as the absence of a biliary stricture requiring restenting at the end of FCSEMS indwell. Stricture recurrence was defined as the need for restenting after stricture resolution, namely recurrence of a previously resolved stricture. Patients were followed until they underwent restenting or until they were stent-free for 5 years, whichever came first. For patients who failed the stent-free status earlier than 5 years after the end of FCSEMS indwell, further treatments were documented in an ad-hoc fashion.

Biliary obstructive symptoms, adverse events, and reinterventions were assessed at 1 week; at 1, 3, 6, and 9 months after FCSEMS placement; at FCSEMS removal; and at 1, 3, 6, 12, 18, 24, 36, 48, and 60 months after FCSEMS removal. Proximal (towards the hilum) or partial distal (in the direction of the duodenum) migration of FCSEMS was assessed by ERCP.

ERCP was performed before and after FCSEMS placement, at FCSEMS removal, and at the time of reported recurrence of biliary obstructive symptoms.

### Statistical methods

Summary statistics were performed to analyze the data for the study, specifically for continuous measurements, mean and standard deviation or median and range was reported and a Wilcoxon rank sum test was used to test between groups where appropriate. For binary data, the rate was reported and a 95% exact confidence interval (CI) was calculated where applicable. Stricture recurrence and freedom from restenting was analyzed using Kaplan-Meier techniques. Univariate and multivariate analyses were used for determining predictors of various endpoints. Specifically, logistic regression using a Firth bias adjustment was used for stricture resolution. For complications and stricture recurrence, a Cox proportional hazards model was performed. Both were performed using the model building technique of step-wise regression, with a *p*-value ≤0.10 to stay in the model and > 0.10 to exit the model. The significance level for all analyses was set at 0.05. All analyses were performed using SAS version 9.4.

## Results

### Patients

Eighteen patients were enrolled between December 2009 and May 2011 at 9 centers in Europe (6), India, Chile, and Canada. Mean age was 53.9 ±13.1 years and 33.3% (6/18) were male. The PCBS was located in the distal (4), mid (6) or proximal (8) bile duct. Most patients (77.8%, 14/18) had prior treatment of the PCBS with one or more plastic stents. All 18 patients completed the study (Table 1).

**Table 1:** Baseline characteristics of patients with post-cholecystectomy biliary strictures

	Summary Statistics
Age	53.9 ±13.1 (18)
Male	33.3% (6/18)
Total Bilirubin Level (mg/dL)	1.6 ±2.0 (17)
Alkaline Phosphate Level (IU/L)	254.9 ±237.8 (17)
Stricture Location	
Distal	22.2% (4/18)
Mid	33.3% (6/18)
Proximal (within 2 cm of the hilum)	44.4% (8/18)
Sphincterotomized	100.0% (18/18)
Any Prior Plastic Stenting History	77.8% (14/18)
Plastic Stents Removed (n) at time of FCSEMS Placement	
1	33.3% (6/18)
2	33.3% (6/18)
4	5.6% (1/18)
Study Stent Size (mm)	
8 × 60	5.6% (1/18)
8 × 80	5.6% (1/18)
10 × 40	16.7% (3/18)
10 × 60	50.0% (9/18)
10 × 80	22.2% (4/18)
Mean Procedure Time (mins)	25.0±16.6 (18)

### Stent placement

The majority of patients (89%, 16/18) received FCSEMS that were 10 mm in diameter. All FCSEMS were placed in trans-papillary position. Stent length was 40 mm (3), 60 mm (10), or 80 mm (5). One stent each were 8 mm × 60 mm and 8 mm × 80 mm in size.

### Shorter-term results

#### Stent removal or migration

Endoscopic FCSEMS removal occurred in 83.3% (15/18) of patients after median indwell of 10.9 months (range 0.9–13.8 months; Table 2). Endoscopic removal was performed at the planned removal time in 11 patients, with median indwell duration of 11.5 months (range 10.4–13.8 months). Early uneventful endoscopic FCSEMS removal was prompted by cholangitis in 4 patients after median indwell duration of 3.8 months (range 0.9–8.9 months).

**Table 2:** Results of endoscopic dilation of post-cholecystectomy biliary strictures with fully-covered self-expanding metal stent (FCSEMS)

	PCBS *N* = 18	95% CI
Endoscopic FCSEMS Removal	83.3% (15/18)	58.6–96.4%
Planned, at 10–12 months	61.1% (11/18)	35.8–82.7%
Early, prior to 10–12 months	22.2% (4/18)	6.4–47.6%
Spontaneous CDM	16.7% (3/18)	3.9–41.4%
Stricture Resolution at End of FCSEMS Indwell	72.2% (13/18)	46.5–90.3%
Overall Adverse Event Rate	38.9% (7/18)	17.3–64.3%

*Abbreviations*: *CDM* complete distal migration, *CI* confidence interval, *FCSEMS* fully-covered, self-expanding metal stent, *PCBS* post-cholecystectomy biliary stricture

In the remaining 3 patients (16.7%) with PCBS, the FCSEMS spontaneously migrated and passed without complications. One of these complete distal migrations (CDMs) was noted at the time of intended removal, 10.8 months. The other two were found at 5.1 and 6.3 months, one suffered from cholangitis and the other had a recurrent stricture. However, in each of these 3 patients the true indwell duration of the FCSEMS is not known.

#### Stricture resolution

**Fig. 3:**
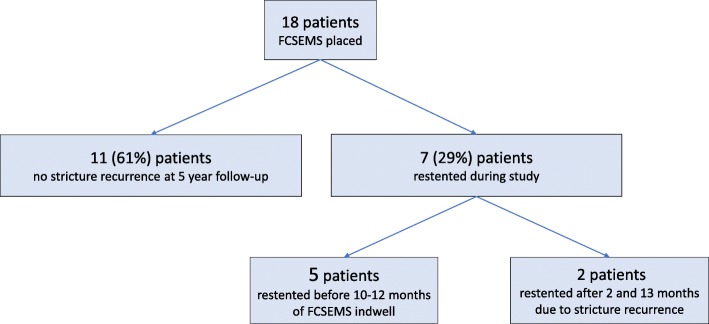
Patient flowchart. The flowchart shows occurrence of post-cholecystectomy biliary strictures resolution after FCSEMS removal and the stricture recurrence at 5 years follow-up in the 13 cases with initial stricture resolution

At the end of FCSEMS indwell, 72% (13/18) of patients had stricture resolution. Of these, 10 patients had FCSEMS indwell duration as planned, 2 underwent early FCSEMS removal due to cholangitis after 59 and 174 days, and 1 had asymptomatic CDM. These 13 patients were considered at risk for stricture recurrence during subsequent follow-up. (Fig. 3).

Excluding the 3 patients with CDM after unknown indwell duration, median FCSEMS indwell duration was 11.3 (range 1.9–13.8) months for the 12 patients with stricture resolution at endoscopic FCSEMS removal vs. 8.9 (range 0.9–10.8) months for the 3 patients without stricture resolution at endoscopic FCSEMS removal (*P* = 0.12). Stricture resolution occurred in 90.9% (10/11) of patients after indwell duration as planned vs. 50% (2/4) after early FCSEMS removal due to cholangitis (*P* = 0.15).

**Fig. 4:**
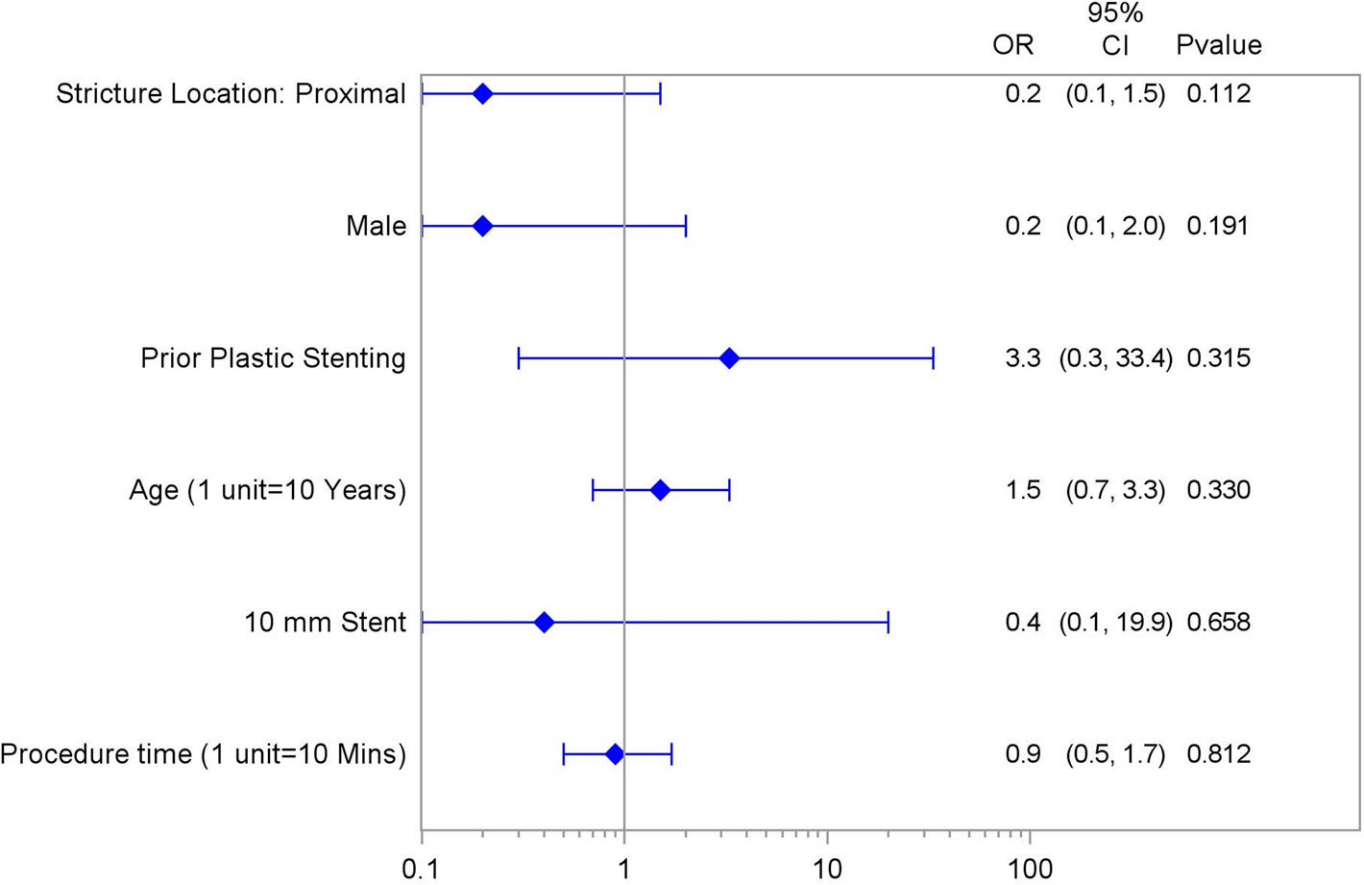
Independent variables by post-cholecystectomy biliary strictures resolution at FCSEMS removal. Univariate Forest Plot of Stricture Resolution at Removal

In a univariate analysis of predictors (including age, sex, proximal stricture location, prior plastic stenting, procedure time, and stent length) for stricture resolution at FCSEMS removal, no significant predictors were found (Fig. 4).

### Longer-term outcomes

#### Stricture recurrence

At 60 months of follow-up, of the 13 patients with stricture resolution at FCSEMS removal or CDM, 2 experienced stricture recurrence, one associated with cholangitis after 2.6 months and one with biliary obstruction after 13.1 months.

**Fig. 5:**
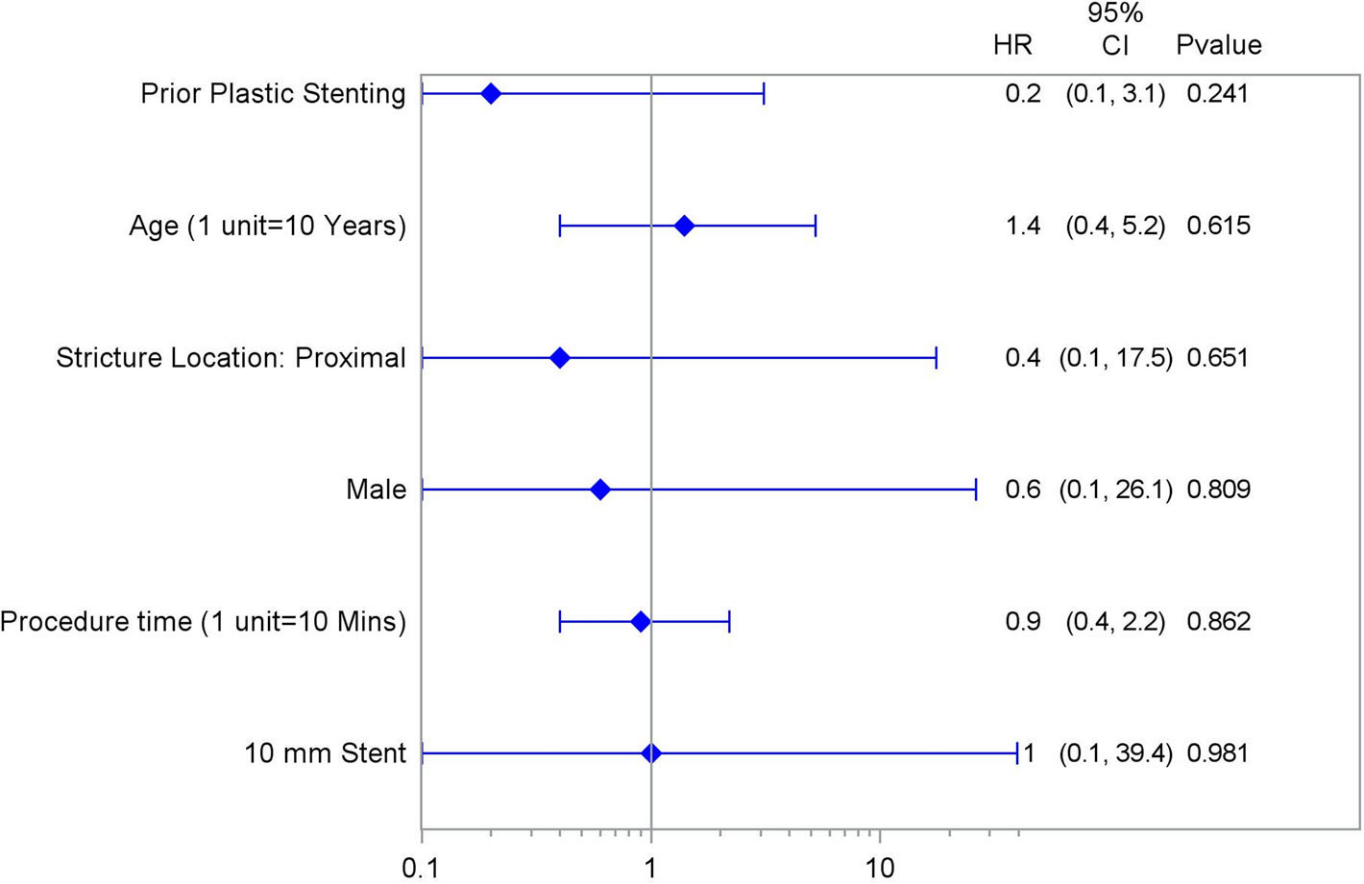
Independent variables by post-cholecstectomy biliary strictures recurrence after initial resolution at FCSEMS removal. Univariate Forest Plot of Stricture Recurrence after Resolution

In a univariate analysis of predictors (including age, sex, proximal stricture location, prior plastic stenting, procedure time, and stent length) for stricture recurrence at FCSEMS removal, no significant predictors were found (Fig. 5).

#### Freedom from Restenting

**Fig. 6:**
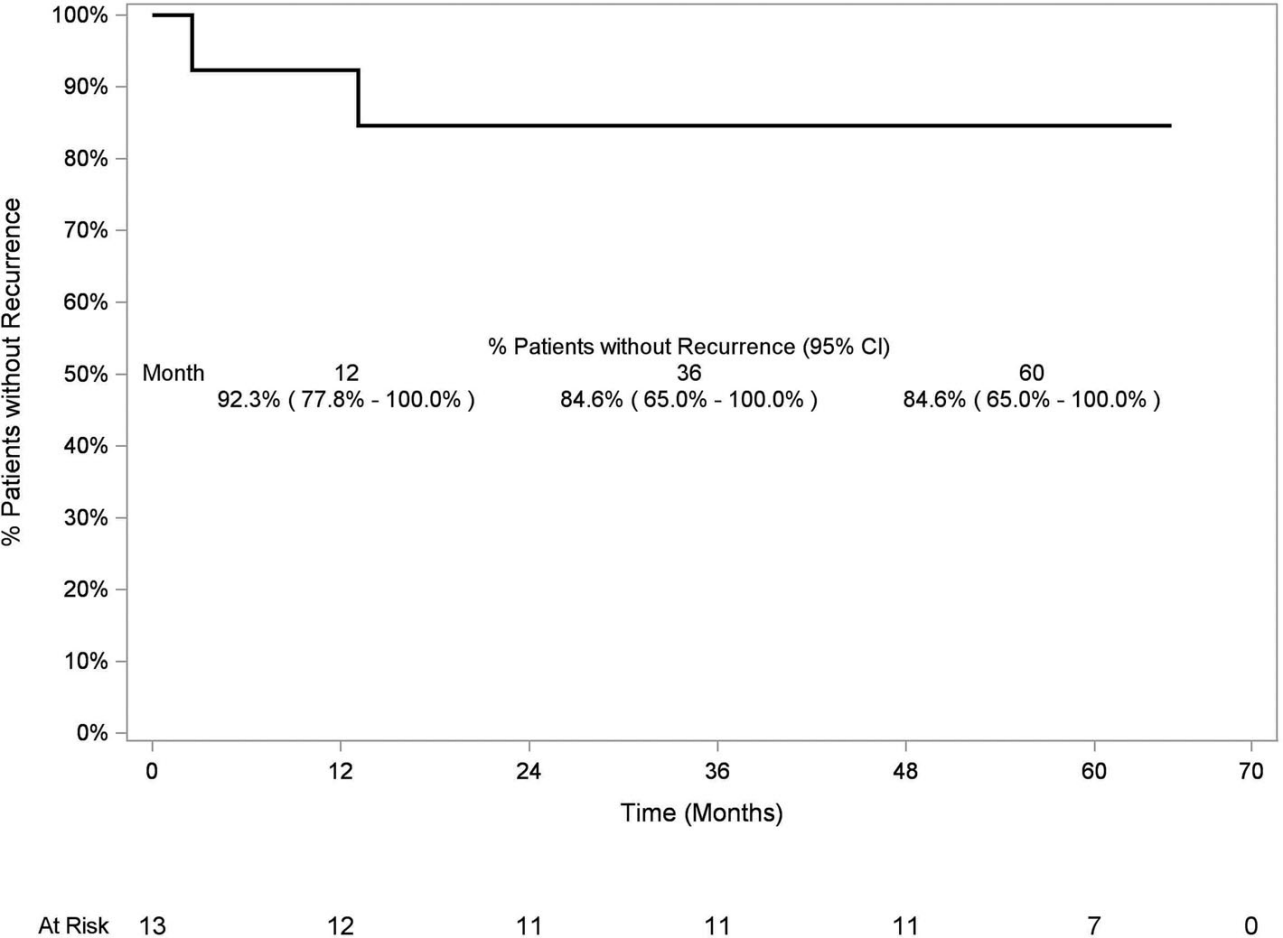
Kaplan-Meier analysis of freedom from post-cholecystectomy biliary strictures recurrence in patients who had stricture resolution at FCSEMS removal. At 60 months after FCSEMS removal, 84.6% (95% CI 65.0–100%) of patients who had stricture resolution at FCSEMS removal remained stent-free

In Kaplan-Meier analysis, at 5 years after FCSEMS removal, the probability of remaining stent-free was 84.6% (95% CI 65.0–100.0%) for patients who had stricture resolution at FCSEMS removal (Fig. 6).

**Fig. 7:**
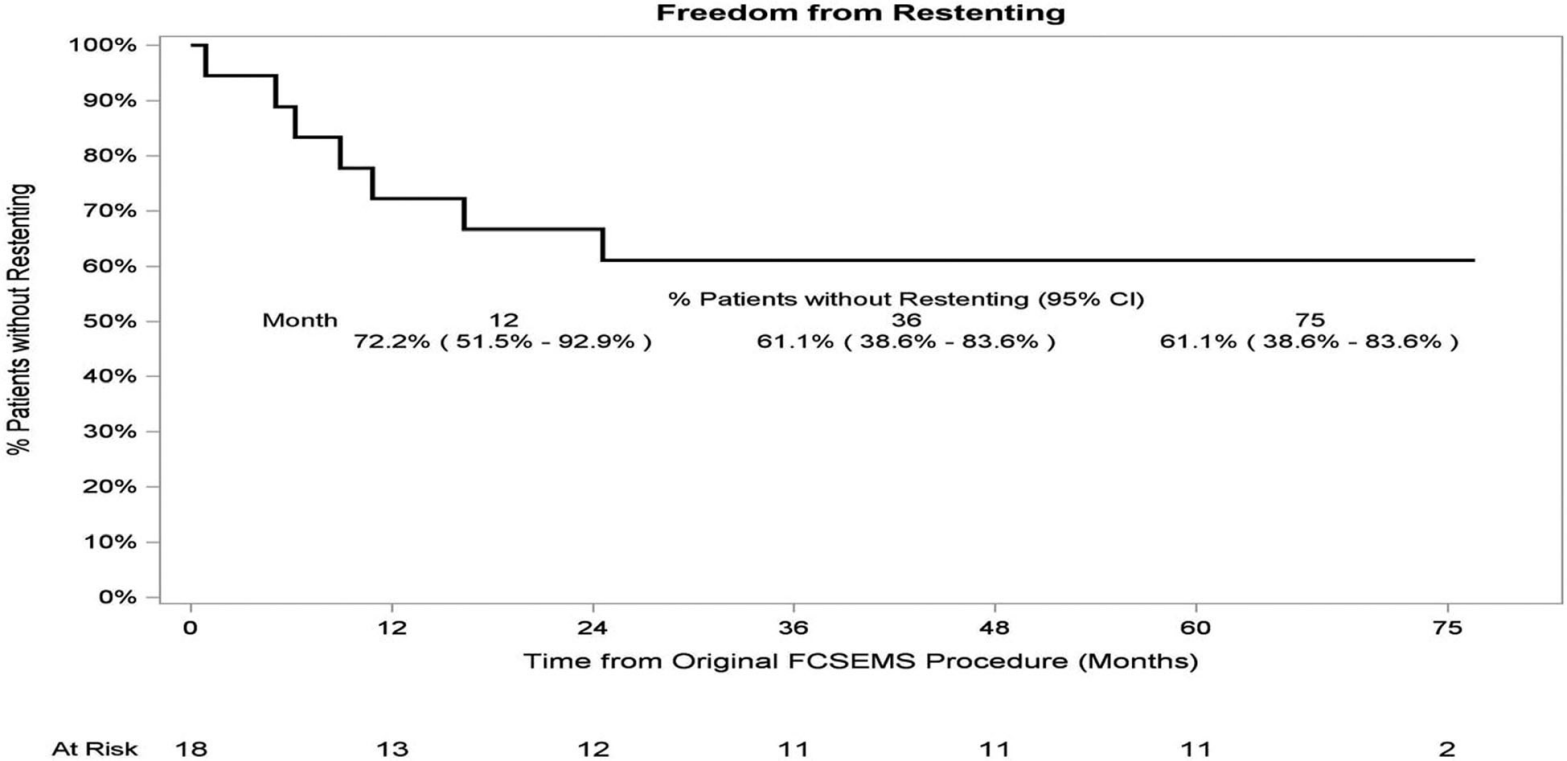
Kaplan-Meier analysis of freedom from restenting in all patients treated for post-cholecystectomy biliary strictures. At 75 months after FCSEMS placement, 61.1% (95% CI 38.6–83.6%) of patients who had FCSEMS placement for treatment of PCBS remained stent-free

In Kaplan-Meier analysis, at 75 months after FCSEMS placement, the probability of remaining stent-free was 61.1% (95% CI 38.6–83.6%) for all patients who had FCSEMS placement for treatment of PCBS (Fig. 7).

### Treatment after stent dysfunction

Throughout the entire course of the study, 7 patients were restented due to loss of stent functionality or recurrence of stricture. Two developed cholangitis leading to early stent removal; 2 had symptoms of biliary obstruction associated with CDM; 1 had unresolved stricture at the time of planned FCSEMS removal; and 2 had recurrence of the stricture after a stent-free period.

All 7 of these patients were restented with plastic biliary stents. Subsequent to plastic restenting, 5 patients underwent 1–3 additional plastic stenting procedures and ultimately were stent-free with normal liver function tests at 5–7 years follow-up after restenting; 1 patient underwent a surgical biliary-enteric anastomosis; and 1 patient was lost to follow-up.

### Adverse events

Stent-related or procedure-related SAEs occurred in 38.9% (7/18) (95% CI 17.3–64.3%) of patients, including cholangitis (6) and pancreatitis (1), and are detailed as follows. Four patients experienced cholangitis that led to early removal of the FCSEMS, 2 of which were immediately restented with plastic stents and 2 remained stent-free for 5 years. One patient developed cholangitis associated with spontaneous CDM of the FCSEMS and was restented with plastic stents. One patient presented with cholangitis at 9.7 months after FCSEMS placement followed by FCSEMS removal as planned after 10.8 months of indwell, without stricture resolution; the patient was restented with plastic stents One patient experienced mild post ERCP pancreatitis after FCSEMS removal as planned, which resolved 6 days post-FCSEMS removal without residual effects; the patient remained stent-free for 2.9 months. All SAEs resolved without sequalae.

## Discussion

In this small, prospective, nonrandomized study, 13 of 18 patients with PCBS experienced stricture resolution upon FCSEMS removal or CDM, and at 5 years follow-up, 84.6% (95% CI 65.0–100.0%) of these patients with initial resolution remained stent-free. In Kaplan-Meier analysis, at 75 months after FCSEMS placement, the probability of remaining stent-free was 61.1% (95% CI 38.6–83.6%) for all patients who received an FCSEMS for treatment of PCBS. The overall adverse event rate was 38.9% (95% CI 17.3–64.3%). These results suggest safe and effective treatment of symptomatic PCBS not involving the main hepatic confluence.

Endoscopic removal took place as planned after 10–12 month indwell in 11 patients, early due to cholangitis in 4 patients, and 3 had CDM. Although there was no significant difference in stricture resolution between the groups (90.9% with indwell duration as planned vs. 50% after early removal, *P* = 0.15), the small sample size may have limited the ability to detect a difference in whether the duration of stenting affects stricture resolution (Table 3).

**Table 3:** Results of endoscopic dilation of post-cholecystectomy biliary strictures with fully-covered self-expanding metal stent (FCSEMS) by removal type

	Planned Removal	Early Removal	CDM
Number of patients (n)	11	4	3
Median months of indwell time (range)	11.5 (10.4–13.8)	3.8 (0.9–8.9)	6.2 (5.1–10.8)
Stricture Resolution at End of FCSEMS Indwell (n)	10	2	1
Stent free status at 5 years (n)	8	2	1

*Abbreviations*: *CDM* complete distal migration, *CI* confidence interval, *FCSEMS* fully-covered, self-expanding metal stent

In 1 patient, the FCSEMS was difficult to remove due to the proximal end being embedded in hyperplastic tissue at the level of cholecystectomy clips; the attempted stent removal was stopped and a new FCSEMS was placed inside of the study stent. Both FCSEMSs were removed endoscopically and uneventfully 2 weeks later according to the “stent-in-stent technique” [15]. This patient remained stent-free for 2.5 months, and then had early stricture recurrence maybe related to the development of hyperplasia and subsequent trauma due to difficult FCSEMS retrieval. The rate of cholangitis in our series was high (6/18) but were successfully resolved by medical/endoscopic treatment without any impact on stricture resolution.

Larger series described the use of FCSEMS to dilate BBS, but looking into subgroup analysis these studies included fewer cases of PCBS, compared to our experience [16–19]. Our study also provided longer follow-up than most other published reports. Nonetheless, relatively promising results were demonstrated pertaining to stricture patency [20, 21]. In addition to maintaining bile duct patency and preventing stricture recurrence, endoscopic therapy of BBS reduced morbidity and mortality, as compared with surgical therapy [22]. Typical endotherapy with MPS includes stent exchanges every 3–4 months, resulting in at least 3 procedures per year. In the present study, 11 patients had their FCSEMS removed at the planned timeframe of 10–12 months. When compared with multiple exchanges of plastic stents over the course of 1 year, this indicates that these 11 patients had approximately 2 fewer endoscopic procedures than they would have if treated with plastic stents. FCSEMS can bring the advantage of fewer ERCPs leading to better patient compliance with reduced burdensome endoscopic treatments. Moreover, maximal dilation is reached rapidly after FCSEMS placement compared to a progressive step-wise dilation when inserting MPS in increasing numbers in successive plastic stenting episodes. The potential associated post- FCSEMS placement pain was not observed in the present study. It should also be noted that reaching the same maximal dilation with MPS compared to an 8 mm or 10 mm diameter FCSEMS is not always technically feasible. Accordingly, given the high rate of continued stricture resolution and freedom from restenting in the present study at 36, 48, and even 60 months (Fig. 6), treatment with FCSEMS should be considered a viable long-term therapy for patients with PCBS not involving the main hepatic confluence.

Three patients in the present study experienced CDM. Spontaneous migration may be a possible risk factor for failure of stricture resolution. Of these 3 patients, only 1 had stricture resolution [1, 2]. Antimigration mechanism for FCSEMS to avoid migration seem promising but need further evaluation [1, 2].

Seven patients were restented with plastic biliary stents and 5 underwent repeated ERCPs for stent exchanges. The possibility for endoscopic retreatment of PCBS recurrence represents the key advantage of endotherapy which can be repeated using the same access. One patient underwent a surgical biliary-enteric anastomosis meaning that endoscopy does not preclude future surgical options if needed.

The limitations of our study include a small sample size and the lack of a control group. Larger patient samples specific to PCBS with follow-up durations comparable to that evaluated in our study are expected. Additionally, it should be noted that treatment with FCSEMS is not a panacea for all PCBS and is limited to strictures located > 2 cm from the main hepatic confluence, due to the risk of side-branch occlusion [1, 2].

## Conclusion

In conclusion, this prospective multi-center study indicates that treatment with FCSEMS is effective for maintaining long-term stricture resolution in patients with “non-hilar” PCBS. Randomized controlled trials are needed to better assess the role and the long-term efficacy of FCSEMS compared with MPS in the treatment of PCBS.

## Data Availability

The data, analytic methods, and study materials for this clinical trial may be made available to other researchers in accordance with the Boston Scientific Data Sharing Policy: http://www.bostonscientific.com/en-US/data-sharing-requests.html

## Abbreviations

BBS: Benign biliary strictures
CDMs: Complete distal migrations
CI: Confidence interval
ERCP: Endoscopic retrograde cholangiopancreatography
FCSEMS: Fully-covered self-expanding metal stent
MPS: Multiple plastic stents
PCBS: Post-cholecystectomy biliary strictures
SAEs: Serious adverse events
